# Supplementary material for: FREQ‐NESS Reveals Age‐Related Differences in Frequency‐Resolved Brain Networks During Auditory Recognition and Resting State
Source: Ann N Y Acad Sci. 2026 Jul 30;1562(1):e70349. doi: 10.1111/nyas.70349 (PMC13419915; doi:10.1111/nyas.70349)
Supplement: Supplementary file 1 — Supporting Information: nyas70349‐sup‐0001‐SuppMat.pdf [file NYAS-1562-0-s001.pdf]

Supplementary Material

FREQ-NESS reveals age-related differences in frequency-resolved brain networks during auditory recognition and resting state

Chiara Malvaso [*4,1], Gemma Fernández-Rubio[1], Mattia Rosso[1,5], Elisa Serra[1,2,3], Vera Rudi[1,2,3], Peter Vuust[1], Morten L. Kringelbach[1,2,3], Claudia Testa[4], and Leonardo Bonetti[†1,2,3]

[1] *Center for Music in the Brain, Department of Clinical Medicine, Aarhus University & The Royal Academy of Music, Aarhus/Aalborg, Denmark*
[2] *Centre for Eudaimonia and Human Flourishing, Linacre College, University of Oxford, Oxford, United Kingdom*
[3] *Department of Psychiatry, University of Oxford, Oxford, United Kingdom*
[4] *Department of Physics and Astronomy, University of Bologna, Bologna, Italy*
[5] *IPEM Institute for Systematic Musicology, Ghent University, Ghent, Belgium*

April 14, 2026

S1 Introduction

To evaluate the robustness and generalizability of the FREQ-NESS pipeline ([1, 2, 3]), we conducted a targeted validation analysis. To reduce computational costs, only the first dataset described in the main text was employed. The above-mentioned dataset is also described in [4] and originally comprised 76 participants. Following the exclusion of one individual who failed to complete the experimental task and an additional six participants whose task accuracy fell below 50%, the final sample for the current analysis consisted of 70 participants. Based on self-reported biological sex (gender identity was not assessed

*Corresponding author: chiara.malvaso2@unibo.it
†Corresponding author: leonardo.bonetti@psych.ox.ac.uk

as it was outside the scope of the study) the sample included 31 males and 39 females. Participants were divided into two age-based cohorts: younger adults ($n = 37$; 18 females, 19 males; age 21.89 ± 2.05 years) and older adults ($n = 33$; 21 females, 12 males; age 66.61 ± 5.02 years).

To validate the necessity and impact of the source reconstruction stage, we performed a parallel analysis where GED was applied directly to the sensor-level data. This allowed for a comparison between sensor-space results with source-reconstructed outputs, thereby determining whether the identified spectral features were inherent to the signal or artifacts of the reconstruction process.

Furthermore, to ensure the reliability of the FREQ-NESS framework and to mitigate the risk of over-fitting, we implemented a k-fold cross-validation. This procedure was employed to quantify the generalizability of the extracted components and to rigorously measure potential overfitting within the pipeline.

In addition to the primary validation analyses, the following supplementary figures and tables provide further technical details and robustness checks:

- Detailed specifications of the filter parameters and the frequencies of interest.
- Statistical modeling of the relationship between behavioral metrics (reaction time and accuracy) and frequency-specific explained variance.
- Analysis of secondary and tertiary variance components, including spectral profile visualizations and their morphological correlation with the primary component.
- Stability assessments of the cluster-based permutation tests across varying iteration counts to ensure statistical convergence.

S2 Sensor level analysis

While a primary strength of the FREQ-NESS pipeline is its application to source-reconstructed MEG data for physiologically grounded network estimation [1], we conducted a parallel sensor-level analysis to evaluate the method's robustness and optimality. This analysis follows the pipeline detailed in the Methods section of the main text (Sections 2.1–2.8), with specific modifications to accommodate sensor-space data.

Specifically, we utilized the first dataset described in Section 2.1, maintaining the experimental design, acquisition parameters, and pre-processing steps outlined in Sections 2.2 through 2.4. Differently from the original FREQ-NESS pipeline, source reconstruction was omitted to test the pipeline's performance directly on raw sensor recordings. To ensure consistency with the magnetometer-based source reconstruction used in the primary analysis, the sensor-level approach is limited to the 102 magnetometer channels.

The resting-state epoching (Section 2.5) and filtering (Section 2.7) procedures remained identical, applying a pass-band filter across the 28 frequencies of interest. Finally, Generalized Eigenvector Decomposition (GED) was implemented as described in Section 2.8. The sole difference in this step was the input dimensionality: the covariance matrices were constructed from the 102 magnetometer signals rather than from the reconstructed voxels.
The results of the sensor-level analysis are presented in Figure S1. When compared to the results obtained via the application of GED on source-reconstructed data (see Figure 2a in the main text), the spectral profiles of the first explained variance component appear qualitatively similar across frequencies. However, the absolute magnitude of the explained variance is lower for the sensor-level analysis.To statistically evaluate the consistency between these levels of analysis, we calculated the correlation between the curves derived from sensor-level data and the corresponding curves from source-reconstructed data for the first three components. Prior to this calculation, data were z-score normalized to ensure the comparison reflected the shape of the frequency-dependent behavior rather than absolute power differences. The results, summarized in Table S1, demonstrate high consistency; correlations were robust (minimum $r = 0.592$) and reached statistical significance across all comparisons ($p << 0.05$). This indicates that the source reconstruction process does not introduce artificial spectral effects, as the fundamental frequency-dependent behavior is preserved from the sensor level. Despite this morphological consistency, the reduced explained variance at the sensor level suggests that individual components hold lower relative importance when identified in sensor space. As detailed in the main text, GED maximizes the contrast between a signal covariance matrix ($\mathbf{S}$), representing in the present analysis narrow-band activity, and a reference matrix ($\mathbf{R}$), representing broadband activity. We thus hypothesized that the lower explained variance in sensor space was due to higher similarity between $\mathbf{S}$ and $\mathbf{R}$. To test this, we computed the correlation between $\mathbf{S}$ and $\mathbf{R}$ for each subject at both sensor and source levels. Independent samples t-tests, performed across all 28 frequencies and corrected for multiple comparisons using the False Discovery Rate (FDR) method, confirmed this idea. The resulting corrected p-values (Table S2) show that $\mathbf{S}$ and $\mathbf{R}$ are significantly more correlated in sensor space than in source space across all frequencies. This indicates a smaller discriminative gap between the narrowband and broadband signals at the sensor level. These findings validate the inclusion of source reconstruction in the FREQ-NESS pipeline. By unmixing signal sources, source reconstruction effectively isolates narrowband oscillatory patterns from the broadband background. Furthermore, the high correlation in curve morphology proves that source reconstruction does not introduce mathematical artifacts, but rather enhances and clarifies the physiological networks already present in the raw sensor data.

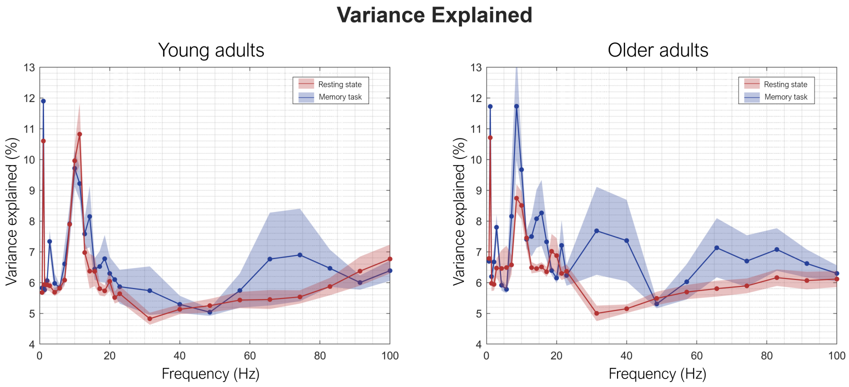

Figure S1: Variance explained by the first component obtained through FREQ-NESS applied at sensor level, shown for young adults (left panel) and older adults (right panel). For both groups, data are presented for resting state (red line) and task (blue line). The plotted lines indicate the mean variance explained across subjects, while the shaded regions denote the standard error of the mean.

Component	Group	Correlation	Significance
1	Memory Old	0.592	9×10^{-4}
	Memory Young	0.703	3×10^{-5}
	Resting Old	0.872	1×10^{-9}
	Resting Young	0.775	1×10^{-6}
2	Memory Old	0.826	6×10^{-8}
	Memory Young	0.790	6×10^{-7}
	Resting Old	0.903	5×10^{-11}
	Resting Young	0.856	6×10^{-9}
3	Memory Old	0.781	1×10^{-6}
	Memory Young	0.686	6×10^{-5}
	Resting Old	0.821	9×10^{-8}
	Resting Young	0.720	2×10^{-5}

Table S1: Morphological consistency between sensor-level and source-level GED components. The table displays Pearson correlation coefficients (r) and associated p-values comparing the z-score normalized spectral profiles across groups and experimental conditions. High correlation values indicate that the frequency-dependent behavior is preserved across analysis levels.

Frequency (f)	FDR p-val
0.714	2.59×10^{-63}
1.071	3.11×10^{-61}
1.429	1.18×10^{-58}
2.143	4.14×10^{-61}
2.857	1.87×10^{-57}
4.286	4.73×10^{-61}
5.714	2.87×10^{-60}
7.143	3.39×10^{-58}
8.571	2.17×10^{-54}
10.000	4.13×10^{-50}
11.428	2.31×10^{-56}
12.857	8.41×10^{-56}
14.285	9.05×10^{-49}
15.714	1.13×10^{-55}

Frequency (f)	FDR p-val
17.142	3.32×10^{-60}
18.571	1.13×10^{-54}
19.999	4.61×10^{-57}
21.428	1.38×10^{-49}
22.856	1.18×10^{-58}
31.427	4.29×10^{-50}
39.998	6.71×10^{-55}
48.569	9.84×10^{-58}
57.140	4.06×10^{-57}
65.711	5.44×10^{-52}
74.282	8.15×10^{-51}
82.853	2.41×10^{-50}
91.424	6.71×10^{-55}
99.995	8.64×10^{-56}

Table S2: Statistical comparison of signal-to-reference similarity across analysis levels. FDR-corrected p-values from independent samples t-tests comparing the correlation between the signal ($\mathbf{S}$) and reference ($\mathbf{R}$) covariance matrices in sensor space versus source space. Significant values across all frequencies indicate a consistently smaller discriminative gap at the sensor level.

S3 Cross validation

Since the primary objective of the GED algorithm is to maximize a user-defined contrast to enhance specific features of the data, there is an inherent risk of overfitting the spatial filters to the specific characteristics of the available dataset at the expense of generalizability. To evaluate the robustness of our proposed approach and ensure that the identified components were not driven by noise, we performed a k-fold cross-validation. A detailed discussion of overfitting within this framework, as well as potential mitigation strategies, can be found in [5].The cross-validation procedure, implemented with $k = 7$, was conducted as follows:

1. For each subject, condition, and frequency, the trials were partitioned into training and testing sets.

2. The training trials were used to compute the covariance matrices, indicated as signal ($\mathbf{S}_{train}$) and reference ($\mathbf{R}_{train}$), which in turn were employed to compute the eigenvalues λ_{train}. For a more detailed explanation of GED see Section 2.8 of the main text.

$$\lambda_{train} = \frac{\mathbf{w}^T \mathbf{S}_{train} \mathbf{w}}{\mathbf{w}^T \mathbf{R}_{train} \mathbf{w}}$$

3. The weights obtained from the training set were then applied to the covariance matrices of the test data to calculate the test eigenvalues:

$$\lambda_{test} = \frac{\mathbf{w}^T \mathbf{S}_{test} \mathbf{w}}{\mathbf{w}^T \mathbf{R}_{test} \mathbf{w}}$$

4. To evaluate the consistency of the results, we compared the eigenvalues for each component and condition across all frequencies and subjects using the following metric to compute the relative difference between λ_{train} and λ_{train}:

$$\%drop = \frac{\lambda_{train} - \lambda_{test}}{\lambda_{train}} \cdot 100 \tag{1}$$

The results of the k-fold validation analysis are presented in Figure S2. For the primary component, the mean relative difference between λ_{train} and λ_{test} was 9.2%, with the distribution skewed toward smaller values (median = 6.5%). For completeness, Figure S2 also illustrates the distributions obtained for the second and third eigenvalues. These components yielded higher mean differences (10.3% and 11.6%, respectively); this trend reflects the fact that successive components capture progressively smaller variance and are, consequently, more driven by noise. Collectively, these results demonstrate a high degree of consistency between λ_{train} and λ_{test}, further supporting the generalizability of the present method.

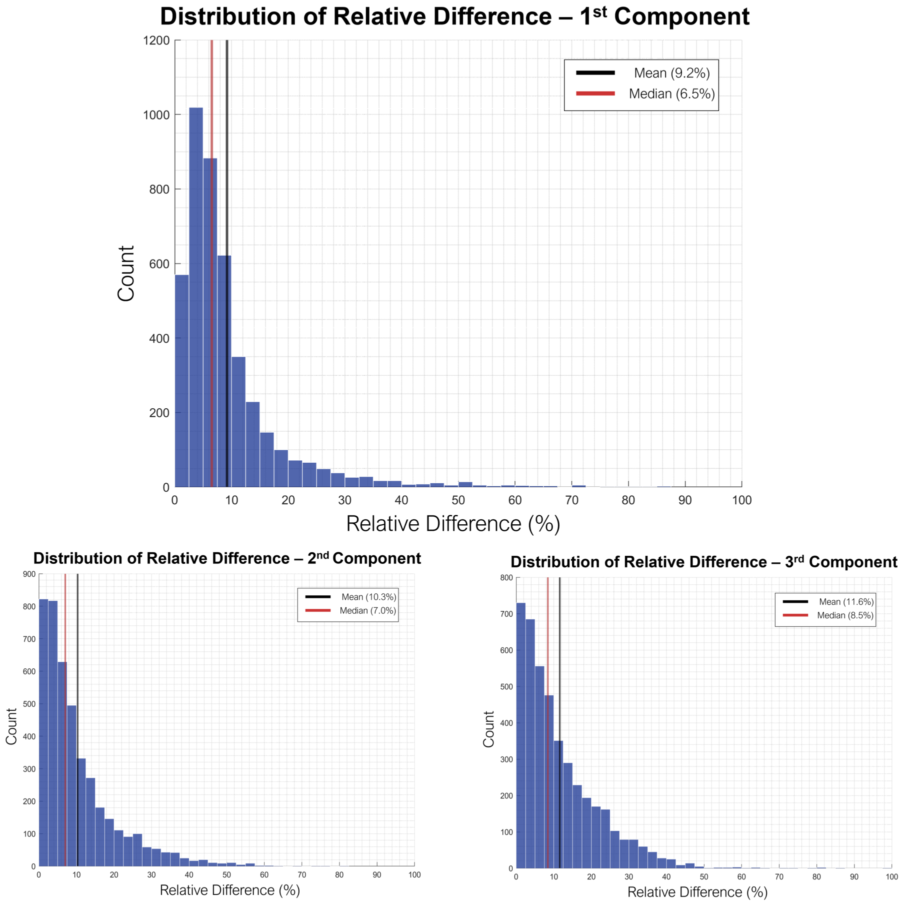

Figure S2: Distribution of relative differences between training and test eigenvalues. The relative difference between λ_{train} and λ_{test} was calculated according to Equation 1. The histograms represent the aggregated relative differences across all subjects, frequencies, and experimental conditions (listening and resting). The upper panel illustrates the distribution for the first component (mean = 9.2%; median = 6.5%), while the lower panel displays the distributions for the second (mean = 10.3%; median = 7.0%) and third (mean = 11.6%; median = 8.5%) components. The consistency observed between training and test eigenvalues reinforces the stability and robustness of the FREQ-NESS approach for event-related analyses.

S4 Supplementary tables

ID	f_C	f_L	f_H	ID	f_C	f_L	f_H
0001	0.714	0.114	1.314	0015	17.142	16.136	18.148
0002	1.071	0.471	1.671	0016	18.571	17.524	19.617
0003	1.429	0.829	2.029	0017	19.999	18.911	21.087
0004	2.143	1.543	2.743	0018	21.428	20.299	22.556
0005	2.857	2.257	3.457	0019	22.856	21.687	24.025
0006	4.286	3.645	4.926	0020	31.427	30.014	32.840
0007	5.714	5.033	6.395	0021	39.998	38.341	41.655
0008	7.143	6.421	7.864	0022	48.569	46.669	50.469
0009	8.571	7.808	9.334	0023	57.140	54.996	59.284
0010	10.000	9.196	10.803	0024	65.711	63.323	68.099
0011	11.428	10.584	12.272	0025	74.282	71.650	76.914
0012	12.857	11.972	13.741	0026	82.853	79.977	85.729
0013	14.285	13.360	15.210	0027	91.424	88.305	94.543
0014	15.714	14.748	16.679	0028	99.995	96.632	103.358

Table S3: Summary of selected frequencies of interest (f_C) and their respective lower (f_L) and upper (f_H) pass-band filter cutoff values. As detailed in Section 2.7, frequency intervals are non-linearly spaced to prioritize the high-density sampling of lower frequencies ($\lesssim$ 20 Hz), which capture more prominent neurophysiological dynamics, while providing broader separation for higher frequency components.

**Model:** $RT \sim$ Variance Explained (Memory) $\times$ Age			
**Frequency [Hz]**	**Source of Variation**	**t-value**	**p-value**
10.0	*Variance Explained*	-3.542	0.015
	Age	-3.013	0.086
	Variance Explained $\times$ *Age*	3.407	0.024

Table S4: Behavioral modeling results relating reaction time (RT) and accuracy to frequency-specific variance explained. P-values are FDR-corrected for multiple comparisons across frequencies. The significant interaction at 10.0 Hz indicates that a more distinct alpha network (higher variance explained) correlates with lower RTs, suggesting increased task readiness.

Models tested in this analysis:

1. $RT \sim$ Variance Explained (Memory) $\times$ Age
2. $RT \sim$ Variance Explained (Resting) $\times$ Age
3. Accuracy $\sim$ Variance Explained (Memory) $\times$ Age
4. Accuracy $\sim$ Variance Explained (Resting) $\times$ Age

Note: Only results from Model 1 reached significance after FDR correction.

Component	Group	Correlation	Significance
2	Memory Old	0.692	5×10^{-5}
	Memory Young	0.645	2×10^{-4}
	Resting Old	0.790	6×10^{-7}
	Resting Young	0.792	5×10^{-7}
3	Memory Old	0.722	1×10^{-5}
	Memory Young	0.868	2×10^{-9}
	Resting Old	0.785	8×10^{-7}
	Resting Young	0.805	2×10^{-7}

Table S5: correlations between the spectral profiles of additional components and the first component. For each experimental cohort and condition (Older and Younger adults, Memory and Resting-state), the frequency-dependent explained variance of the second and third components was correlated with the corresponding profile of the first component (presented in the main text). As indicated by the high correlation coefficients and associated significance levels, successive components exhibit strong morphological consistency with the primary component. This similarity suggests that while the secondary and tertiary components reinforce the stability of the observed effects, they do not contribute distinct spectral information. These results support the selection of the first component, corresponding to the largest eigenvalue, as the primary representative of the data, consistent with established methodological recommendations [5].

S5 Supplementary figures

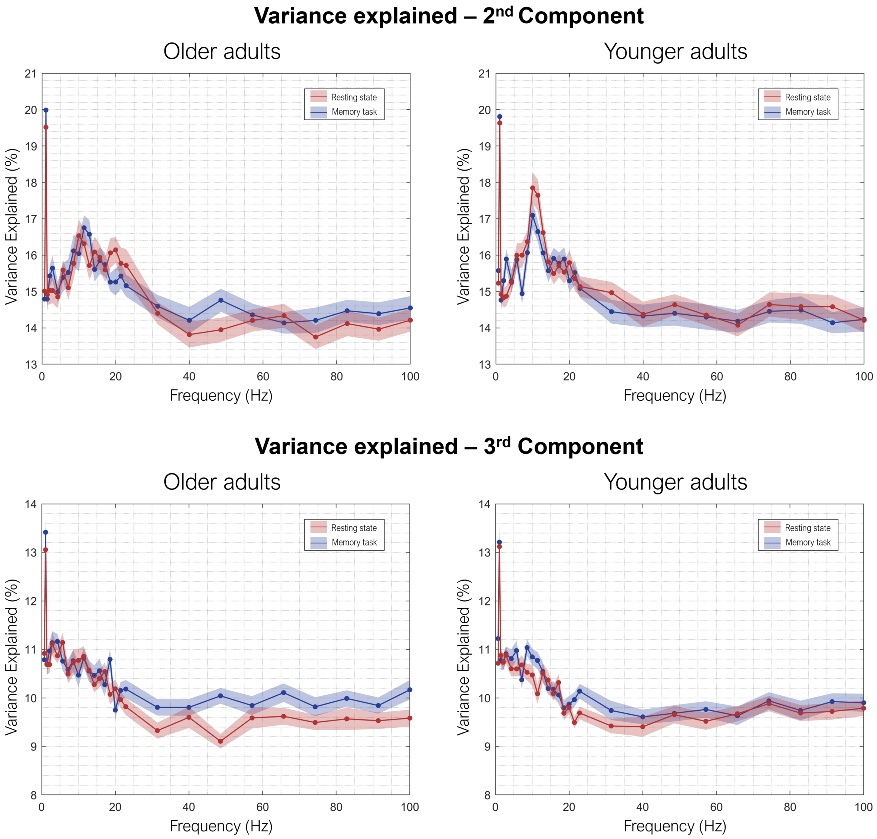

Figure S3: Spectral profiles of explained variance for secondary and tertiary components. The plots illustrate the variance explained as a function of frequency for the second (top) and third (bottom) components across both older and younger adult groups. Both components exhibit frequency-dependent behavior qualitatively similar to that of the first component described in the main text. Visual inspection, corroborated by the correlation analysis in Table S5, confirms the stability and robustness of the network patterns identified by the FREQ-NESS pipeline. The high degree of consistency across components demonstrates that the primary effect is well-preserved, while the higher absolute magnitude of the first component validates its use as the optimal descriptor of the underlying physiological activity.

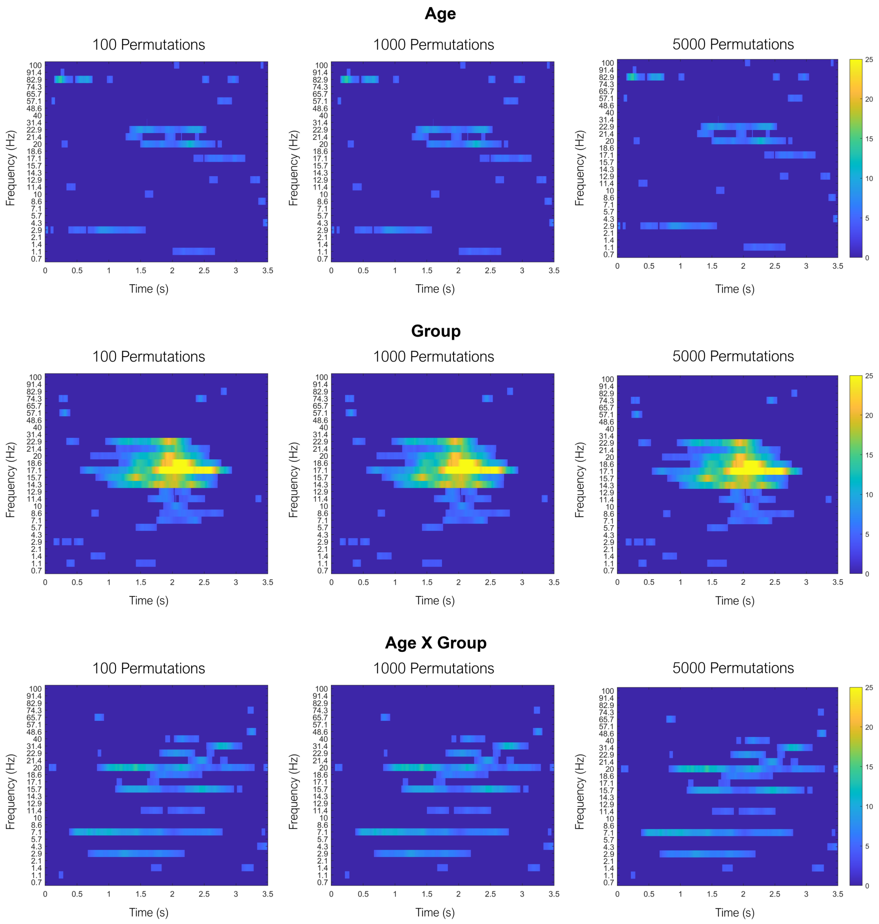

Figure S4: Stability analysis of the cluster-based permutation test. The panels display F-statistics derived from the ANOVA performed on the power time series, highlighting significant effects of condition, age, and their interaction. To enhance visual clarity, F-values associated with non-significant effects (i.e., those falling outside identified significant clusters) were thresholded to zero. The columns illustrate the results obtained using $N = 100$ (left), $N = 1000$ (center), and $N = 5000$ (right) permutations. The frequency and temporal extent of the significant clusters remains invariant across the different permutation counts, indicating that 100 permutations provide sufficient statistical stability for these data while minimizing computational time. Detailed descriptions of the cluster formation strategy and significance testing are provided in Section 2.9.2 of the main text.

References

[1] M. Rosso, G. Fernández-Rubio, P.E. Keller, et al. "FREQ-NESS Reveals the Dynamic Reconfiguration of Frequency-Resolved Brain Networks During Auditory Stimulation". In: *Advanced Science (Weinheim)* (2025). Published online April 10, 2025. DOI: 10.1002/advs.202413195.

[2] Kenneth Shinozuka et al. "LSD reconfigures the frequency-specific network landscape of the human brain". In: *bioRxiv* (2025). DOI: 10.1101/2025.03.21.644645. eprint: https://www.biorxiv.org/content/early/2025/03/24/2025.03.21.644645.full.pdf. URL: https://www.biorxiv.org/content/early/2025/03/24/2025.03.21.644645.

[3] Kenneth Shinozuka et al. "Ibogaine is associated with reorganization of high-beta brain networks in veterans with post-traumatic stress disorder". In: *bioRxiv* (2026). DOI: 10.64898/2026.03.20.713241. eprint: https://www.biorxiv.org/content/early/2026/03/24/2026.03.20.713241.full.pdf. URL: https://www.biorxiv.org/content/early/2026/03/24/2026.03.20.713241.

[4] L. Bonetti, G. Fernández-Rubio, and M. Lumaca. "Age-related neural changes underlying long-term recognition of musical sequences". In: *Communications Biology* 7 (2024), p. 1036. DOI: 10.1038/s42003-024-06587-7. URL: https://doi.org/10.1038/s42003-024-06587-7.

[5] Michael X Cohen. "A tutorial on generalized eigendecomposition for denoising, contrast enhancement, and dimension reduction in multichannel electrophysiology". In: *NeuroImage* 247 (2022), p. 118809. ISSN: 1053-8119. DOI: https://doi.org/10.1016/j.neuroimage.2021.118809. URL: https://www.sciencedirect.com/science/article/pii/S1053811921010806.
